# Refractoriness about adaptation

**DOI:** 10.3389/fnhum.2015.00038

**Published:** 2015-02-04

**Authors:** Robert P. O'Shea

**Affiliations:** ^1^Discipline of Psychology, School of Health and Human Sciences, Southern Cross UniversityCoffs Harbour, NSW, Australia; ^2^BioCog, Institute of Psychology, University of LeipzigLeipzig, Germany

**Keywords:** EEG, ERP, neural adaptation, neural fatigue, predictive coding, repetition suppression, stimulus specific adaptation, visual mismatch negativity (vMMN)

“Let us go down, and there confound their language, that they may not understand one another's speech.”

Genesis 11:7 (King James Version, 1611).

Recently Stefanics et al. (2014) reviewed the visual (v) mismatch negativity (MMN), a negative shift in voltage of an event-related potential (ERP) to an unpredictable, rare, *deviant* stimulus in a regular sequence of identical, *standard* stimuli. Stefanics et al. have written a wonderfully comprehensive review of the vMMN, concluding that it might be a sign of predictive coding (Mumford, 1992; Friston, 2005; Winkler and Czigler, 2012).

I have two comments:

1. Stefanics et al. referred to one explanation of the MMN, needing to be distinguished from true predictive coding, as “refractoriness.” I argue that a better term for refractoriness is “adaptation.”
2. Stefanics et al. (2014) said the MMN debate about adaptation, as defined above, was “not particularly productive” (p. 2), although they did concede that it needs to be taken into account. I argue that adaptation ought to be harmonized into any complete MMN explanation.

I should emphasize that I have no quibble with the logical necessity of distinguishing sluggishness of repeatedly stimulated neurons from the MMN and that this has been achieved numerous times, as Stefanics et al. have recorded.

## Etymology and meaning of refractoriness and adaptation

By “refractoriness,” Stefanics et al. meant a “neurophysiological effect reflecting neuronal ‘fatigue’” (p. 3) or “synaptic depression due to the depletion of vesicles from the presynaptic terminal” (p. 9). They gave other names for the “response attenuation … (from repeated presentations of a standard, including) repetition suppression, stimulus-specific adaptation (SSA), [and] habituation” (p. 3), although these are not strictly synonyms.

O'Shea (2015) showed that “refractoriness” is common in the MMN (and ERP) literature but rare in related literatures.

Refractoriness comes from Latin *refractarius*: stubborn or obstinate—its meaning in ordinary language—and is different from its MMN meaning—tired. The physiological meaning of the adjective “refractory” refers to the state of a neuron or cardiac nerve after electrical activity begins for which it is impossible to generate more, no matter how intense the stimulation (the absolute refractory period) or for which it very difficult to generate more (the relative refractory period) (e.g., Hodgkin, 1948; Chapman, 1966). For neurons, these refractory periods are of the order of milliseconds—much too short to be responsible for the refractoriness supposed to underlie the MMN. Moreover, synaptic depletion occurs only at stimulation rates much higher than typically used in vMMN studies, over 10 Hz (Fernández-Alfonso and Ryan, 2004).

There is also a general slowing of responses, such as key presses, when people are given a task to perform shortly after another—the psychological refractory period (Welford, 1952), But this is not from fatigue of neurons, but likely from a central bottleneck and serial preparation of responses (Pashler, 1994).

Adaptation comes from Latin *adaptare*: to fit. In ordinary language it means to make something suitable for a new purpose. In cognitive neuroscience, it means a change in the responsiveness of neurons to fit them to the range of current inputs (Webster, 2012). Adaptation is not a defect of neurons but something that has been designed by evolution to ensure survival. It likely involves some active process, because some neurons of sensory pathways do not adapt (Ohzawa et al., 1985; Solomon et al., 2004).

## Adaptation and the MMN

One view of predictive coding is that it is an epistemic approach aimed at *why* cognitive-neuroscience phenomena occur, sitting comfortably with approaches that are aimed at mechanistic explanations—at *how* such phenomena occur (such as via adaptation) (Garrido et al., 2008, 2009). To restrict understanding of adaptation to neural fatigue or to see adaptation as opposed to the MMN could limit the richness of understanding that viewing phenomena from two perspectives can yield.

The predictive-coding explanation is that the brain constructs predictions of future sensory input from past sensory input and matches these against actual input, generating an error signal when the input is different from the prediction. This occurs at different levels of the brain, each one involving more and more abstract properties of sensory input.

This is rather similar to the epistemic role of adaptation: to alter the responsiveness of neurons based on past input to ensure they are maximally responsive to the range of inputs. It occurs at different levels of the brain, each one involving more and more abstract properties of sensory input. For example, in the visual system, cones adapt to the prevailing light level to operate over more than three orders of magnitude of light intensity (Valeton and Van Norren, 1983). Retinal ganglion cells encode contrast and they show contrast adaptation, allowing for meaningful signals whatever the range of contrasts in the visual field (Solomon et al., 2004). They also adapt to more complex properties of the visual scene, such as orientation and spatiotemporal modulations (Hosoya et al., 2005). Cortical neurons adapt to the properties they encode, such as orientation, spatial frequency, and motion (Clifford et al., 2007). Inferotemporal cortical neurons adapt functionally to the shapes of stimuli presented anywhere in their receptive fields (De Baene and Vogels, 2010).

Studies suggesting that adaptation needs to be harmonized with MMN explanations include:

- An extensive theoretical treatment (May and Tiitinen, 2010).
- Unpredictablity's reducing adaptation (Summerfield et al., 2008; Kok et al., 2014).
- Stefanics et al.'s conclusion that stimulus-specific adaptation (Nelken and Ulanovsky, 2007) is a possible neural substrate for MMN.
- Musall et al.'s (2014) study showing that mechanical stimulation of rats' whiskers resulted in the rats' being behaviorally more sensitive to a deviant stimulus than when they stimulated the rats' cortices optogenetically and perceptually identically, bypassing cortical and downstream adaptation.

## Conclusion

Using “refractoriness,” a term that is essentially unknown in fields such as fMRI, animal models, and psychophysics, creates a Tower of Babel. I believe it is better in science if one's language unites, rather than divides. Replacing “refractoriness” in the MMN vocabulary with adaptation terms and searching for a rapprochement between adaptation and MMN could bring considerable explanatory benefits.

### Conflict of interest statement

The author declares that the research was conducted in the absence of any commercial or financial relationships that could be construed as a potential conflict of interest.
